# AI-Assisted Optical Coherence Tomography Segmentation for Enhanced Diagnosis of Inherited Retinal Diseases

**DOI:** 10.1167/tvst.14.12.8

**Published:** 2025-12-04

**Authors:** Virginie G. Peter, Michel Hayoz, Davide Scandella, Raphael Sznitman, Pascal Escher, Martin S. Zinkernagel

**Affiliations:** 1 Department of Ophthalmology, Inselspital, Bern University Hospital, Bern, Switzerland; 2 ARTORG Center, University of Bern, Bern, Switzerland; 3 Department for BioMedical Research, University of Bern, Bern, Switzerland; 4 Bern Photographic Reading Center, Department of Ophthalmology, Inselspital, Bern University Hospital, Bern, Switzerland

**Keywords:** inherited retinal disease (IRD), optical coherence tomography (OCT), artificial intelligence (AI)

## Abstract

**Purpose:**

Inherited retinal diseases (IRDs) are rare and diverse, posing a diagnostic challenge in ophthalmology. This study aimed to determine whether artificial intelligence (AI)-assisted image processing can improve IRD diagnosis and provide insights into disease characteristics. We used an optical coherence tomography (OCT) segmentation algorithm to characterize retinal features in IRDs. Two control groups were included to enhance the contextual understanding of these features: healthy eyes and eyes with age-related macular degeneration (AMD). An AI-driven classification model was then used to classify the data into disease and control groups.

**Methods:**

We analyzed 327 images from 181 patients with IRD and 146 control individuals, including healthy subjects and patients with AMD. IRD cases were stratified into macular and retinal dystrophies. Automated segmentation of six retinal layers and detection of nine biomarkers were performed on retinal OCT images using the AI-based RetinAI Discovery tool. A random forest classifier differentiated macular IRD, retinal IRD, and controls.

**Results:**

The model detected IRD with 91% accuracy and achieved 91% accuracy in differentiating macular from retinal IRD. Key OCT features for differentiation included reduced perifoveal photoreceptor and outer nuclear layer thicknesses and increased retinal nerve fiber layer thickness in retinal IRD. Macular IRD featured significant foveal photoreceptor and outer nuclear layer thinning.

**Conclusions:**

This study shows that standardized OCT image analysis combined with AI-based classification can accurately detect and stratify IRDs. The model's high accuracy highlights its potential as a reliable diagnostic tool in ophthalmology.

**Translational Relevance:**

This AI-assisted OCT evaluation approach enhances ophthalmic diagnostics by improving IRD detection and classification.

## Introduction

Inherited retinal diseases (IRDs) are a diverse group of vision-impairing disorders, including several heterogeneous clinical types, presenting a diagnostic challenge in ophthalmology. Affecting approximately 1 in 4000 individuals, they represent a leading cause of vision impairment and blindness in the working-age population.^1^^,^^2^ This raises the question of whether artificial intelligence (AI)-assisted optical coherence tomography (OCT) readings—a powerful and rapidly advancing method—could benefit the field, a possibility we explore and discuss in the following sections. Diagnosing IRDs is particularly complex due to their rarity and the broad spectrum of clinical subtypes, which often have overlapping phenotypic characteristics. In practice, healthcare professionals encounter these conditions infrequently, leading to prolonged diagnostic journeys for affected individuals, involving multiple referrals and inconsistent access to specialized care.^3^ Furthermore, the diagnostic process is hindered by the lack of standardized diagnostic criteria, leading to variability in clinical approaches and outcomes. This underscores the need for simple diagnostic tools and approaches to improve systematicity in diagnosing IRDs.^4^

Accurate diagnosis for these conditions classically relies on a comprehensive clinical assessment, including medical and ocular history, family history, complete ophthalmological examination, and imaging modalities such as OCT and fundus autofluorescence (FAF), as well as electrophysiological testing. Additionally, molecular genetic testing is essential in confirming diagnoses and informing affected subjects on personalized treatment strategies. These represent resource-intensive assessments, which rely on interpretation by specialized ophthalmologists with years of experience. However, such experts are confined to dedicated centers that are not uniformly accessible across different regions, leading to significant variation in diagnostic outcomes. A diagnostic aid based on a detailed yet universally available imaging modality like OCT thus represents an attractive solution to facilitate initial assessment and thereby improve accessibility to referral centers for individuals affected with IRD.

IRDs can be categorized into retinal and macular subtypes, based on the affected region of the retina. This classification provides a straightforward and clinically useful framework to facilitate diagnosis and guide further assessments, also providing insights into the expected visual outcomes of the condition. In this article, we focus on two major classes of IRD, the retinal and the macular IRDs. Retinal IRDs are generalized photoreceptor diseases typically identified by abnormal full-field electroretinography (ERG) among which retinitis pigmentosa (RP) is a prototypical example, characterized by the primary degeneration of rod photoreceptors followed by cone loss. This progression results in symptoms such as night blindness, visual field constriction, and eventually central visual impairment. In contrast, macular IRDs, affect the macular region specifically. Stargardt disease is the most common example in this category, often presenting with a normal full-field ERG. Symptoms primarily include reduced central vision, color vision abnormalities, photophobia, and nystagmus.

Advancements in imaging are transforming ophthalmology by improving retinal visualization. Large datasets improve understanding of rare diseases like IRDs, whereas AI-assisted analysis promises better diagnostic accuracy and efficiency. However, challenges remain, including the need for automated OCT segmentation and disease monitoring markers, and AI models capable of handling variability in disease progression and phenotype expression.^4^ Spectral-domain OCT, with its noninvasive nature and accessibility, enables objective, automated IRD evaluations, allowing precise measurements and biomarkers’ identification, such as hyper-reflective foci or intraretinal fluid. Although retinal imaging-based diagnostics have advanced, most studies focus on common retinal diseases rather than IRDs.^5^^–^^11^ With a focus on IRD, algorithms have been developed using color fundus or FAF imaging to aid IRD classification, but they lack the ability to visualize retinal layers and detect subtle structural changes.^12^^–^^15^ Few studies have exploited OCT to stratify IRDs, but interpreting these images remains challenging due to the heterogeneous presentation of IRDs.^16^^,^^17^ Using a combination of imaging modalities could enhance stratification accuracy but integrating and interpreting multimodal data are complex and require extensive computational resources. The inconsistency in the availability of all required imaging modalities across different clinical settings also limits the practicality of such approaches.^18^^,^^19^

The aim of this study is to evaluate the performance of an existing AI-based OCT image processing tool in detecting IRDs, classifying them into macular and retinal types, and describing the characteristics of these two types. Using the existing automated OCT feature extraction tool RetinAI Discovery, retinal layer thickness and biomarker presence were analyzed in a cohort of 181 patients with IRD. Additionally, an AI-driven classification model was introduced to identify IRD cases and categorize them into retinal and macular subtypes.

## Methods

### Study Cohort and Clinical Information and OCT Acquisition Modalities

This was a retrospective analysis of anonymized macular OCT images obtained from patients who visited the University Hospital of Bern, Bern, Switzerland, between August 2008 and March 2023, and had provided written informed consent for the use of anonymized data for scientific purposes. This study was approved by the ethics committee of our institution (2023-00768) and adhered to the principles of the Declaration of Helsinki. A total of 327 OCT volume scans from 327 subjects were included, including individuals affected by IRDs (*n* = 181) and healthy subjects (*n* = 57). Additionally, a control cohort consisting of 89 OCT images from eyes with neovascular age-related macular degeneration (nAMD), randomly selected from our internal database was included. These eyes met the inclusion and exclusion criteria of the RIVAL study, the most important of which was a visual acuity letter score of 23 or greater.^20^ Clinical IRD diagnoses were established by careful and standardized ophthalmological and genetic evaluations as part of our clinical consultations. Subjects with various IRD subclasses were included and assigned to one of two categories on the basis of their clinical diagnosis (phenotyping): retinal IRD or macular IRD*.* The retinal IRD group comprised 93 cases with a median age at initial presentation of 39 years (range = 4 to 76 years), diagnosed with RP, Usher syndrome, Bardet–Biedl syndrome, “neuropathy, ataxia, and RP” (NARP) syndrome, Bietti crystalline corneoretinal dystrophy, and rod-cone dystrophy. The macular IRD group included 88 subjects with a median age of 41 years (range = 6 to 81 years), affected with Stargardt macular dystrophy, macular dystrophy, occult macular dystrophy, cone dystrophy, Best’s vitelliform dystrophy, and cone-rod dystrophy (Table 1, results section). Patient data, including age, ophthalmological history, as well as OCT volume data from one eye acquired at the initial visit, were included. Macular OCT volume scans were acquired with a Spectralis spectral domain OCT (SD-OCT) imaging system (Heidelberg Engineering, Inc., Heidelberg, Germany) with the following resolutions: 496 × 512 × 49 (*n* = 317), 496 × 1024 × 49 (*n* = 4), and 496 × 768 × 61 (*n* = 7). OCT scans covering an area of at least 5.50 mm × 5.50 mm and less than 10.0 mm × 10.0 mm were included. In each case, the right or left eye was randomly chosen, so that one OCT from a single eye was considered per individual.

**Table 1.** Summary of OCT Counts, Diagnoses, and Patient Age Distribution

Category	Number of OCT Volumes	Diagnoses Included in Category	Age (Median, Range)
Healthy	57	–	56 y, [10–81 y]
Retinal IRD	93	Retinitis pigmentosa [76], Usher [10], Bardet–Biedl syndrome [3], NARP [2], Bietti crystalline corneoretinal dystrophy [1], Rod-cone dystrophy [1]	39 y, [4–76 y]
Macular IRD	88	Stargardt macular dystrophy [35], Macula dystrophy [26], Occult macular dystrophy [10], Cone dystrophy [8], Best Vitelliform Dystrophy [5], Cone-rod dystrophy [4]	41 y, [6–81 y]
AMD	89	Neovascular type	n/a

### Macular OCT Morphological Feature Detection and Quantification

Automatic segmentation of six retinal layers, retinal fluids, and detection of nine AMD-related biomarkers was performed on retinal OCT images using the AI-based platform Discovery version 2.2 (RetinAI AG, Switzerland).^21^^–^^23^ Note that this tool had been initially developed and optimized for characterizing OCTs in AMD but has since proven useful for other macular pathologies.^24^^,^^25^ Auto-segmented retinal layers included the following: retinal nerve fiber layer (RNFL); ganglion cell layer + inner plexiform layer (GCL + IPL); inner nuclear layer + outer plexiform layer (INL + OPL); outer nuclear layer (ONL) + external limiting membrane; photoreceptor outer segments + retinal pigment epithelium (PR + RPE); chorio-capillaries + chorio-septae (CC + CS); and retinal total thickness (RT). Segmented fluids included subretinal fluid (SRF), intraretinal fluid (IRF), and pigment epithelium detachment (PED). There was no systematic assessment of retinal layer segmentation and fluid detection accuracy. However, an experienced ophthalmologist visually reviewed the central horizontal OCT image in half of the randomly selected cases and in all the misclassified samples, ensuring segmentation quality by verifying that layers or fluid accumulations were detected and their borders reasonably matched an unzoomed glance. Detected biomarkers included: SRF; IRF; hyper-reflective foci (HF; diameter 25 µm to 50 µm); DRUSEN, hyper- (and/or hypo-)reflective areas between the RPE and Bruch's membrane; reticular pseudodrusen (RPD); drusenoid deposits between the photoreceptors and above the RPE, typically an undulation of the ellipsoid zone; epiretinal membrane (ERM); geographic atrophy (GA); outer retinal atrophy (ORA); and fibrous pigment epithelium detachment (FPED).^23^ Retinal thicknesses are given in micrometers, and percent change was calculated as (*thickness_in_disease – thickness_in_healthy*) / (*thickness_in_healthy*) * 100 – where thickness_in_healthy represents the mean retinal layer thickness of the 57 healthy subjects. Retinal thickness measurements are provided for three concentric rings centered around the fovea, as defined in the Early Treatment Diabetic Retinopathy Study (ETDRS) study: the foveal circle (1 mm diameter), the parafoveal ring (extending 3 mm around the fovea), and the perifoveal ring (extending 6 mm around the fovea). To compare retinal thicknesses between pathological groups and a healthy reference, a 2-sample Welch’s *t*-test was performed on mean retinal thickness values, with *P* values less than 0.01 considered statistically significant. Biomarkers are a classification output per B-scan, and the mean occurrence rate per volume (C-scan) was computed as follows: biomarker detection produced a metric ranging from 0 to 1, where, simply put, 0 indicates no detection and 1 indicates detection in all slices of the macular OCT volume. Details of its calculation are published elsewhere and can be summarized as follows.^23^ First, the probability of the presence of a given biomarker in each B-scan p_i_ is obtained and converted to a binary variable c_i_ according to the equation below:

$$\begin{array}{c} c_{i}\left\{\begin{array}{c} 1\;\;\;\;for\;\;\;\;p_{i}>0.5\\ 0\;\;\;\;for\;\;\;\;p_{i}\leq 0.5 \end{array}\right. \end{array}$$

Then, the mean occurrence rate of a biomarker denoted *cbar* is calculated for each OCT volume.

### Classification

Given the available sample size and feature dimensionality of the dataset, a random forest classifier was chosen to differentiate retinal from macular IRD and controls.^26^ Healthy and AMD “controls” were predicted as distinct classes but merged for assessing classifier performance and precision/recall calculations, simplifying the classification of IRD versus non-IRD cases, in line with the study's proof-of-concept focus on broad disease categories. As a result, IRD and AMD were treated as mutually exclusive. Utilizing retinal thicknesses and biomarker occurrence rates as input features, the classifier was configured with 100 trees and a minimum of 2 samples per leaf to prevent overfitting. To mitigate bias toward the majority class, the class weights were adjusted inversely proportional to class frequencies during training. We used four-fold cross-validation, ensuring a class-balanced split to evenly distribute samples across pathological groups in each fold. Performance evaluation relied on accuracy, precision, and recall metrics. Additionally, feature importance scores (FIS) within the random forest were utilized to assess the relative contributions of individual features to predictions. FIS for a feature was computed as the normalized product of the number of times it split a node and the samples it affected, ensuring a sum of one for all scores. The larger the score, the more important the feature is perceived to be.

## Results

### Characteristic Changes in Retinal Layer Thickness in IRD: A Comparison Between Retinal and Macular IRD

**Figure 1.**
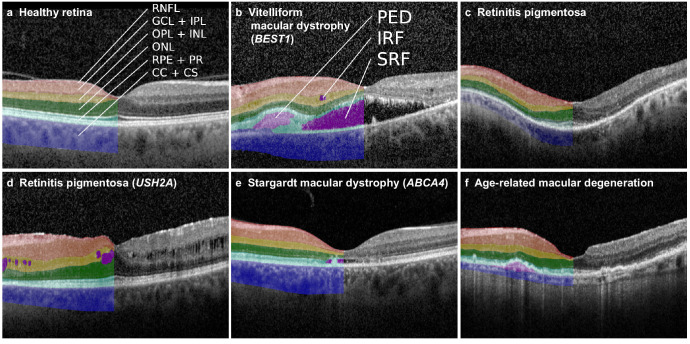
**Automated retinal layer segmentation and detection of biomarkers in OCT volumes.** Examples of a healthy retina (**a**), typical IRD subtypes (**b–d**), and age-related macular degeneration are shown. (**b**) A case of *BEST1*-associated vitelliform macular dystrophy characterized by sub- and intra-retinal fluid, a fibrous PED and hyper-reflective foci. (**c**) A case of retinitis pigmentosa showing peri- and para-foveal atrophy of the outer retinal layers. (**d**) A *USH2A*-associated retinitis pigmentosa case, with ONL atrophy, intra-retinal fluid, and epiretinal membrane. (**e**) A case of *ABCA4*-associated Stargardt macular dystrophy, featuring foveal atrophy of the photoreceptor and outer nuclear layers. The automated segmentation algorithm interprets this as subretinal fluid and detects hyperreflective foci in both the inner and outer retinal layers. CC + CS, chorio-capillaries + chorio-septae; GCL + IPL, ganglion cell layer + inner plexiform layer; INL + OPL, inner nuclear layer + outer plexiform layer; ONL, outer nuclear layer + external limiting membrane; PED, pigment epithelium detachment; IRF, intraretinal fluid; SRF, subretinal fluid; PR + RPE, photoreceptors + retinal pigment epithelium; RNFL, retinal nerve fiber layer.

From a cohort of retinal and macular IRD cases, along with controls, we obtained the structural characteristics of macular OCT images from the first visit using an automated retinal layer and fluid segmentation and biomarker detection tool (see Table 1). Visual inspection of selected cases revealed adequate segmentation of retinal layers and delineation of sub/intra-retinal fluid, despite the altered retinal architecture observed in various IRD diagnoses (Fig. 1).

**Figure 2.**
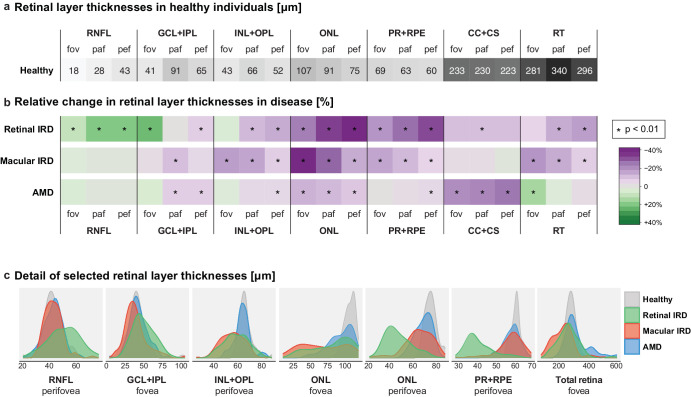
**Retinal layer thickness in control and disease groups.** (**a**) Mean thicknesses (in µm) at the fovea (fov), parafovea (paf), and perifovea (pef) in healthy subjects (*n* = 57). (**b**) Mean percent change in retinal layer thickness in retinal IRD (*n* = 93), macular IRD (*n* = 88), and AMD (*n* = 89), with *green* indicating thickening and *purple* indicating thinning. Significant differences from the healthy reference (*P* < 0.01, *t*-test) are *asterisked*. Changes in retinal thickness highlight characteristic disease-associated changes accentuated in the perifovea for retinal IRD and at the fovea in the case of macular IRD. (**c**) Density curves showing the distribution of retinal layer thicknesses in selected layers. In retinal IRD, a noticeable thickening of the RNFL layer and thinning of the ONL and PR + RPE layers are seen at the perifovea.

As a reference, we calculated the average thicknesses of the retinal layers in healthy controls (Fig. 2a). The relative change in thicknesses of the different retinal layers in disease was calculated, and a statistical test identified which layers were significantly altered compared to the healthy reference, as shown in Figure 2b. Numbers and *P* values are given in Supplementary Figure S1.

In retinal IRDs, the averaged changes generally became gradually more pronounced from the fovea to the perifovea, that is, the outer macular ring. There was a significant thickening of the RNFL layer throughout the entire macular area and of the GCL + IPL layer at the fovea. Such thickening of the innermost retinal layers appeared as a unique feature of retinal IRD. The ONL, which contains the photoreceptor nuclei, showed the most pronounced percentage of thinning, followed by thinning of the PR + RPE layer. The GCL + IPL layer was significantly reduced only at the perifovea, whereas the CC + CS layer was reduced in the parafoveal area. Total retinal thickness was significantly reduced outside the foveal center. Altogether, the retinal IRD class included a relatively homogeneous group of diagnoses displaying similar OCT features, notable for a consistent thinning of the outer retinal layers, particularly the ONL and photoreceptor outer segments, especially at the perifovea (Fig. 2c).

In macular IRD, the greatest relative change in thickness was observed at the ONL, more severely centrally than peripherally. Despite being the retinal layer with the most thinning on average, some cases of macular IRD had surprisingly preserved ONL thickness (see Fig. 2c). This variability was interpreted in the context of high diversity within the macular IRD category. Indeed, diseases like occult macular dystrophy or non-Stargardt macular dystrophy could present with preserved outer retinal layers in early stages, whereas conditions like cone and cone-rod dystrophy typically showed marked ONL and PR + RPE layer reduction earlier in the disease. The PR + RPE and INL + OPL layers were significantly reduced across the whole macular area, whereas the GCL + IPL layer showed significant thinning parafoveally. Total retinal thickness was reduced over all areas considered. There was no significant thinning of the CC + CS layer in macular IRD, contrasting with nAMD, where this layer was the most significantly altered throughout the macula.

In nAMD cases, there was significant thinning of the ONL layer in all macular regions, making ONL thinning a consistent feature across the retinal diseases examined here. However, the pattern of thinning distinguished IRD subtypes and AMD, being more pronounced peripherally in retinal IRD and centrally in macular IRD, whereas it was even throughout the whole macula in AMD. Pan-macular RP + RPE thinning, a consistent feature in IRDs, was only significant in the perifovea in AMD. Other changes in AMD included thinning of the GCL + IPL layer para- and peri-centrally and thinning of the INL + OPL layer at the perifovea. In terms of total RT, AMD showed a significant thickening at the fovea without significant thinning elsewhere, contrasting with IRDs. A detailed look at the distribution curve of total RT at the fovea shows a bimodal distribution of AMD cases, corresponding to cases with and without retinal thickening, attributable to intra- or sub-retinal fluid and/or fibrosis (see Fig. 2c). There was also a significant reduction in choriocapillaris thickness across all macular areas in AMD. This choroidal thinning tended to be present in retinal IRD, although to a lesser extent, but was not observed in macular IRDs.

### Detection of OCT Biomarkers in IRD

**Figure 3.**
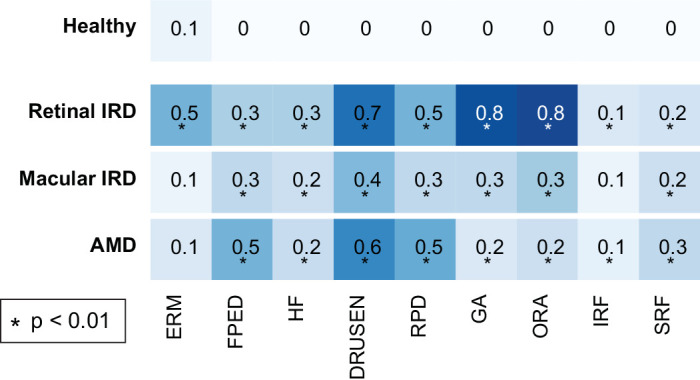
**Detection of** **nine** **OCT biomarkers comparing controls to retinal IRD, macular IRD, and AMD.** Values represent the mean detection rates in macular volumes, ranging from 0 (not detected; depicted as *light blue*) to 1 (present throughout the entire macular volume; depicted as *dark blue*). Biomarkers significantly enriched in disease groups (*P* < 0.01) are *asterisked*.

We evaluated nine AMD-related biomarkers across macular OCT volumes and conducted a statistical test to compare their levels in diseased versus healthy controls (Fig. 3). As expected, all biomarkers were absent in the healthy cohort, except for the ERM, which averaged 0.1, indicating a subtle ERM finding in the OCT of a healthy individual with an average age of 56 years. ERM was not increased in macular IRD and AMD compared with controls but showed a marked increase in the retinal IRD cohort. The HF biomarker, representing small hyper-reflective points throughout any retinal layer, was increased on average in all diseases considered, and more so in retinal IRD. The DRUSEN and RPD biomarkers, indicating deposits below and above the RPE, respectively, were detectable to varying extents in all diseases considered. The average degree of outer retinal atrophy (GA, ORA) was high. in IRD and present to a milder degree in the other diseases considered. IRF was slightly increased on average in retinal IRD and AMD but not in macular IRD. SRF was increased on average in all three disease entities, with a slightly higher prevalence in AMD.

In summary, in retinal IRD, all biomarkers showed significant detection compared to the healthy group, with particularly notable levels of ERM, DRUSEN, and atrophy of the outer retinal layers, along with the presence of FPED and HF. In macular IRD, most biomarkers were significantly increased, except for ERM and IRF. In AMD, all biomarkers except ERM were significantly detected, with less atrophy of the outer retinal layers than in retinal IRD but more FPED.

### AI-Based Classification of Retinal IRD, Macular IRD, and Controls

**Figure 4.**
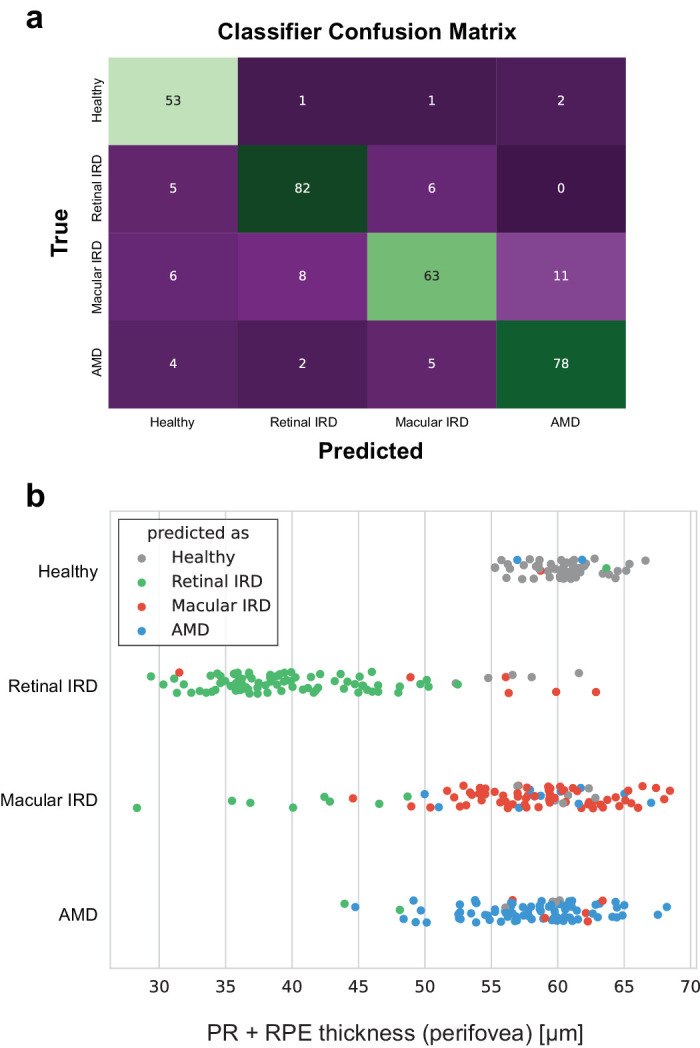
**Classification of retinal and macular IRD versus controls.** (**a**) Aggregated classifier confusion matrix across all folds. Diagonal elements show the number of correctly classified samples, whereas off-diagonal elements indicate the number and predicted class of misclassified samples. (**b**) Plot of photoreceptors + retinal pigment epithelium (PR + RPE) thickness against class predictions. Each *dot* represents a sample: *green dots* in the macular IRD row are misclassified as retinal IRD, typically late-stage cases.

To evaluate the effectiveness of OCT-derived information in differentiating macular IRD from retinal IRD, we used a random forest classifier to categorize the images into these two groups, as well as two control groups. Our results demonstrated an overall classification accuracy into 4 groups of 0.86 ± 0.02, indicating the feasibility of predicting classes based solely on macular OCT scans (Table 2; Fig. 4a). More specifically, detecting the presence of IRD (any class) versus aggregated controls was achieved with an accuracy of 0.91, whereas differentiating macular IRD from retinal IRD was achieved with an accuracy of 0.91. Precision, which reflects the proportion of correctly identified instances of a class among all predicted instances of that class, was calculated as 0.89 ± 0.05 for retinal IRD, whereas recall, measuring the classifier’s ability to correctly identify all relevant instances of a class, was 0.88 ± 0.06. For macular IRD, precision and recall were 0.84 ± 0.07 and 0.72 ± 0.05, respectively. Controls exhibited precision of 0.87 ± 0.06 and recall of 0.94 ± 0.02.

**Table 2.** Results and Confusion Matrix

Group	Precision	Recall	Accuracy
Control	0.87 ± 0.06	0.94 ± 0.02	
Macular IRD	0.84 ± 0.07	0.72 ± 0.05	
Retinal IRD	0.89 ± 0.05	0.88 ± 0.06	
Overall			0.86 ± 0.02

**Table 3.** Feature Importance (Top 10)

Feature	FSI
PR + RPE perifoveal	0.087
PED parafoveal	0.081
DRUSEN	0.074
ORA	0.059
FPED	0.059
RPD	0.058
PED foveal	0.049
ONL perifoveal	0.047
GA	0.041
ONL parafoveal	0.040

Similar precision and recall values between controls and retinal IRD indicate a balanced performance in correctly identifying these classes. However, slightly lower recall for macular IRD suggests that the model may have missed identifying some instances of this category. Analysis of misclassified cases revealed that performance often relied on the stage of disease progression (Fig. 4b). For instance, early stage retinal IRD may manifest in the mid-periphery without affecting the macular region covered by OCT, leading to misclassification as healthy. Conversely, late-stage macular IRD cases may involve the perifoveal region, making them challenging to distinguish from cases of retinal IRD. Additionally, differentiation between macular IRD and AMD pathological groups is particularly challenging due to similarities in early-stage features.

As anticipated, we observed that the perifoveal PR + RPE thickness emerged as the most influential feature (FIS = 0.087) (see Table 3). This finding aligns with the distinct pattern of PR + RPE thicknesses perifoveally observed in retinal IRD, which was more reduced than in the other pathologies considered (see Fig. 2c). The notable influence of this feature on the classifier also explains why the earliest stage retinal IRD cases could be misclassified as healthy or macular IRD, as depicted in Figure 4b. The FIS are evenly distributed among the features, indicating that most features contribute comparably to the overall classification.

## Discussion and Conclusions

We aimed to investigate the feasibility of developing a fully automated classification tool for IRD based solely on OCT images. This initiative was driven by the necessity for more efficient and accessible diagnostic methods for rare retinal diseases like IRD. Our study utilizes a commercially available tool to segment retinal layers and detect biomarkers, which forms the basis for an AI-based classification of IRDs.

The first part of this report documents the alterations observed in IRDs in a detailed and interpretable manner. It specifically highlights distinctive OCT changes characterizing retinal and macular IRDs, informing not only about the disease, but also the criteria behind AI-based classification. Among the relevant findings, thickening of the inner retinal layers was identified as a unique feature of retinal IRD, a previously described phenomenon related to neuronal-glial remodeling in response to outer retinal atrophy.^27^ The ONL, followed by the PR + RPE layer, showed the most significant thinning in IRD, consistent with the known histopathologic degenerative process, in which the layers containing the photoreceptor outer segments and nuclei become thinner or completely atrophied in the late stages.^28^^–^^30^ Our observations also highlighted that the pattern of thinning, whether peripherally or centrally accentuated or homogeneous, distinguishes IRD subtypes from one another and from AMD. Additionally, we noted that significant reductions in choroidal thickness observed in AMD were absent in macular IRD. Choroidal thickness changes in IRDs have been overlooked in the past, and recent OCT-based studies have produced inconsistent results. Our findings suggest the potential of integrating choroidal thickness evaluations in future IRD assessments, which could be clinically relevant, for instance, in helping differentiate cases of Stargardt disease masquerading as AMD or pattern dystrophy.^29^^,^^31^ Although these findings align with long-standing knowledge, the quantitative comparison of OCT layer thicknesses in these two IRD entities with AMD, as presented in this report, offers a novel and more comprehensive perspective.

Regarding biomarkers, several findings emerged. We found epiretinal membranes with increased prevalence in retinal IRD, but not in macular IRD or AMD, which is consistent with previous literature reporting that up to 36% of individuals affected with RP have epiretinal membranes due to preretinal glial cell proliferation and/or secondary to inflammation.^32^^–^^34^ Hyper-reflective foci were detected in all the diseases considered, particularly retinal IRD, although the underlying pathomechanism differs among entities.^29^ The detection of hyper-reflective foci represents an area for further refinement with clinical relevance, as evidence from previous reports in RP suggests that their abundance and localization within specific retinal layers may correlate with disease stage, severity, and the condition of the RPE and PR layers, as well as visual acuity.^34^^,^^35^

FPED, DRUSEN, and RPD are primarily associated with AMD and typically used to characterize OCT findings in AMD, yet, in this study, they were surprisingly also detected in IRD. It is important to clarify that, in a simplistic way and in the context of the AI-based tool utilized in this study, FPED indicates an undulating RPE, RPD indicates an undulating ellipsoid zone, and DRUSEN denote small subretinal foci.^23^ These manifestations can also appear in IRD from different underlying pathogenic mechanisms. Indeed, various changes occur in the outer retinal layers in these diseases, including pigmentary clumping or migration, focal thickenings of the PR and/or RPE layers, deposition of debris from degenerated photoreceptors, and formation of outer retinal tubulations. These can manifest as undulations or interruptions of the outer retinal layers, flecks, atypical drusen-like lesions, as well as sub- or outer retinal hypo-/hyper-reflective foci.^29^^,^^31^ Depending on the affected layer, these findings may be correctly detected by the AI-based tool but may be, in the context of IRD, inaccurately labeled as FPED, DRUSEN, and/or RPD. Detection of these signs remains relevant and has proven useful in the subsequent step of AI-based diagnostic classification of IRDs. This, while highlighting a limitation in our study, mainly underscores the importance of these subtle OCT signs in IRD. If specifically trained and optimized for IRD, a tool dedicated to the detection of such biomarkers could be relevant in distinguishing IRD types; to our knowledge, no such tool is currently available.

In a second step, we evaluated the utility of the OCT-derived data described above in distinguishing between macular and retinal IRDs using AI. Our findings indicate a good overall classification accuracy of 0.86 ± 0.02 for distinguishing among retinal IRD, macular IRD, and controls. Precision and recall metrics further elucidated the model’s performance, with retinal IRD and controls showing slight better precision and recall than macular IRD, implying challenges in its differentiation from other entities, potentially due to the larger heterogeneity within this group. Analysis of misclassified instances further highlighted complexities in distinguishing early-stage retinal IRD from macular IRD or healthy, and late stage macular IRD from retinal IRD. Particularly influential was the perifoveal PR + RPE thickness in differentiating retinal IRD from other pathologies. Overall, our results underscore the feasibility and nuances of using OCT and AI for IRD classification, while revealing areas needing further refinement, especially in distinguishing macular IRD from other conditions like AMD.

Some limitations of this study should be discussed. First, the OCT feature extraction tool used was primarily optimized for AMD. Second, the number of patients affected by IRD is limited. Consequently, developing a dedicated biomarker detection tool for IRD is appealing but requires a larger training dataset than was available in this study. Retinal layer segmentation was less limiting as layers and fluids appeared to be well delineated despite the modified retinal structures in IRD, although a systematic performance assessment of segmentation was not performed. We acknowledge that this is an important limitation, as understanding the failure modes would provide valuable context for future large-scale analysis and improve the reliability of the segmentation process. Moreover, segmenting the PR + RPE as a single layer was a limitation because these two layers individually represent some of the most critical information. Recent developments of Discovery include the ability to differentially segment these two layers separately, promising better resolution and potentially improving classification performance in the future. Another limitation is the broad categorization of IRD into retinal and macular types rather than the specific diagnoses. While detailed results on OCT characteristics for each IRD diagnosis (e.g., Stargardt macular degeneration, occult macular dystrophy, cone dystrophy, …) would be appealing, this lay outside our scope due to the limited number of samples, risking rather inaccurate conclusions. Better performances can be anticipated from larger, more diverse datasets. Despite these limitations, a remarkable level of diagnostic accuracy was achieved. This highlights a real potential for OCT-based diagnostic tools in IRD, and anticipates improvements from larger, more diverse datasets, and addressing the limitations discussed above. Specifically, we see the following as the key next steps, some of which would become feasible with a larger, more diverse IRD-specific dataset. Re-training the model to classify IRDs at a more granular level could help account for variability across subtypes, molecular etiologies, and disease stages. Integrating multi-modal imaging, such as FAF, could further enhance classification performance and provide a more comprehensive understanding of IRD. Additionally, an optimized OCT feature extraction tool, trained and validated specifically for IRDs—including comparisons with expert assessments—would be essential. Future studies could explore regression-based approaches rather than classification to better account for overlapping diagnoses, such as IRD and AMD, and enhance model flexibility. Last, direct comparison of the AI model's accuracy with expert ophthalmologists is essential to validate its real-world utility.

In summary, this study systematically characterizes OCT changes in a real-world IRD cohort, addressing a previously unexamined gap. It demonstrates that a two-step process in which OCT images are first characterized by an established tool, and then classified based on the obtained features, enables accurate stratification of IRD into categories while providing a detailed description of OCT changes. This enhances the interpretability of such models for better acceptance and use in clinical settings. Indeed, many AI models, particularly deep learning, often operate as black boxes, making it difficult for clinicians to understand the decision making process. Looking ahead, integrating automated assessments derived from OCT imaging into clinical practice holds significant promise. Such advancements could enable real-time evaluation of retinal images, providing standardized and objective feedback to eye care professionals. Ultimately, this could support more timely and accurate clinical decision making, leading to improved outcomes for patients with IRD.

## Supplementary Material

Supplement 1
