# Supplementary material for: AI-Assisted Optical Coherence Tomography Segmentation for Enhanced Diagnosis of Inherited Retinal Diseases
Source: Transl Vis Sci Technol. 2025 Dec 4;14(12):8. doi: 10.1167/tvst.14.12.8 (PMC12697681; doi:10.1167/tvst.14.12.8)
Supplement: Supplement 1 [file tvst-14-12-8_s001.pdf]

## Change in retinal layer thicknesses

* $p < 0.01$

| RNFL | fov | paf | pef |
|---|---|---|---|
| Retinal IRD | +1.6µm<br>+8.9%<br>p=8.42e−04<br>* | +5.5µm<br>+19.5%<br>p=6.89e−13<br>* | +8.5µm<br>+20%<br>p=6.72e−08<br>* |
| Macular IRD | +0.4µm<br>+2.1%<br>p=3.99e−01 | +0.1µm<br>+0.5%<br>p=8.41e−01 | +0.5µm<br>+1.3%<br>p=6.39e−01 |
| AMD | +0.6µm<br>+3.4%<br>p=1.04e−01 | +0.3µm<br>+1%<br>p=6.55e−01 | +0.4µm<br>+0.9%<br>p=7.36e−01 |

| GCL+IPL | fov | paf | pef |
|---|---|---|---|
| Retinal IRD | +9.4µm<br>+23.3%<br>p=7.08e−05<br>* | −0.2µm<br>−0.2%<br>p=9.36e−01 | −3.9µm<br>−6%<br>p=5.46e−03<br>* |
| Macular IRD | −1.1µm<br>−2.6%<br>p=6.61e−01 | −8.5µm<br>−9.4%<br>p=6.19e−05<br>* | −2.4µm<br>−3.7%<br>p=5.24e−02 |
| AMD | +2.1µm<br>+5.2%<br>p=3.31e−01 | −5.9µm<br>−6.5%<br>p=6.33e−04<br>* | −4.7µm<br>−7.2%<br>p=2.13e−05<br>* |

| INL+OPL | fov | paf | pef |
|---|---|---|---|
| Retinal IRD | +1.8µm<br>+4.2%<br>p=3.35e−01 | −7.2µm<br>−10.8%<br>p=1.54e−07<br>* | −7.7µm<br>−14.7%<br>p=8.61e−18<br>* |
| Macular IRD | −7.6µm<br>−17.7%<br>p=7.19e−05<br>* | −16%<br>−10.7µm<br>p=8.08e−14<br>* | −6.7%<br>−3.5µm<br>p=3.49e−06<br>* |
| AMD | +0.8µm<br>+1.9%<br>p=6.56e−01 | −2.1µm<br>−3.2%<br>p=3.82e−02 | −2.2µm<br>−4.2%<br>p=1.41e−04<br>* |

| ONL | fov | paf | pef |
|---|---|---|---|
| Retinal IRD | −25.1µm<br>−23.5%<br>p=5.73e−13<br>* | −30.4µm<br>−33.5%<br>p=1.63e−29<br>* | −27.7µm<br>−36.8%<br>p=1.47e−43<br>* |
| Macular IRD | −39.7µm<br>−37.1%<br>p=1.94e−19<br>* | −25.3µm<br>−27.8%<br>p=1.66e−19<br>* | −10.3µm<br>−13.6%<br>p=1.40e−10<br>* |
| AMD | −13.1µm<br>−12.2%<br>p=8.38e−09<br>* | −10.2µm<br>−11.2%<br>p=2.18e−11<br>* | −6µm<br>−7.9%<br>p=1.66e−08<br>* |

| PR+RPE | fov | paf | pef |
|---|---|---|---|
| Retinal IRD | −12.3µm<br>−17.8%<br>p=1.52e−13<br>* | −16.1µm<br>−25.7%<br>p=2.83e−30<br>* | −19.3µm<br>−32.1%<br>p=1.53e−46<br>* |
| Macular IRD | −11µm<br>−16%<br>p=2.23e−07<br>* | −7.2µm<br>−11.4%<br>p=1.64e−08<br>* | −3.2µm<br>−5.2%<br>p=3.07e−04<br>* |
| AMD | −0.8µm<br>−1.2%<br>p=6.17e−01 | −1.5µm<br>−2.4%<br>p=1.13e−01 | −2.4µm<br>−4.1%<br>p=7.57e−05<br>* |

| CC+CS | fov | paf | pef |
|---|---|---|---|
| Retinal IRD | −22.2µm<br>−9.5%<br>p=1.08e−02 | −22.6µm<br>−9.8%<br>p=9.21e−03<br>* | −22.4µm<br>−10%<br>p=1.09e−02 |
| Macular IRD | −6.2µm<br>−2.7%<br>p=4.18e−01 | −3.8µm<br>−1.6%<br>p=6.10e−01 | +0.9µm<br>+0.4%<br>p=9.06e−01 |
| AMD | −46.7µm<br>−20%<br>p=3.93e−08<br>* | −49.1µm<br>−21.3%<br>p=6.07e−09<br>* | −53.3µm<br>−23.9%<br>p=2.22e−10<br>* |

| RT | fov | paf | pef |
|---|---|---|---|
| Retinal IRD | −15.7µm<br>−5.6%<br>p=8.54e−02 | −45.5µm<br>−13.4%<br>p=9.00e−11<br>* | −49µm<br>−16.6%<br>p=2.52e−20<br>* |
| Macular IRD | −51.3µm<br>−18.2%<br>p=1.05e−07<br>* | −49µm<br>−14.4%<br>p=3.10e−12<br>* | −18.2µm<br>−6.1%<br>p=1.33e−05<br>* |
| AMD | +43.2µm<br>+15.4%<br>p=3.75e−05<br>* | +7.1µm<br>+2.1%<br>p=2.69e−01 | −8.3µm<br>−2.8%<br>p=1.96e−02 |

**Supplementary figure 1**: Details in  in numerical values for Figure 2b, including differences in mean retinal thickness from the healthy reference in µm, relative change in %, and p-values determined by Welch's t-test.
